# In vitro comparison of apical leakage in root canals obturated with 0.04 and 0.02 tapered gutta-percha

**Published:** 2010-08-15

**Authors:** Maryam Bidar, Ghazal Sadeghi, Maryam Gharechahi, Mohammad Mortazavi, Maryam Forghani

**Affiliations:** 1 Department of Endodontics, Dental Research Center/Mashad University of Medical Sciences, Mashad, Iran; 2 Endodontist; 3 Endodontist, Dental Research Center/Mashad University of Medical Sciences, Mashad, Iran

**Keywords:** Apical leakage, Filtration, Root canal obturation, Tapered gutta-percha

## Abstract

**Introduction:** Gutta-percha is the most commonly used material for root canal obturation; it has been recently manufactured with different tapers. The aim of this *in vitro* study was to compare microleakage of canals obturated with standard gutta-percha (0.02 taper) or the new 0.04 taper gutta-percha master cone using the cold lateral condensation technique.

**Materials and Methods:** Forty-four extracted single rooted teeth were selected. The crowns were removed and all the canals were prepared using RaCe rotary files. The teeth were then divided into experimental (n=2) and control (n=2) groups. In the first study group, the teeth were obturated with 0.02 taper gutta-percha master cone and lateral condensation. In the second study group, the canals were obturated by 0.04 tapered master cones and the same obturation method. The degree of leakage was measured using fluid filtration method. Data were analyzed statistically by student t-test.

**Results:** There was no significant difference between the mean microleakage of two experimental groups (P=0.558).

**Conclusion:** Lateral condensation technique using 0.04 tapered master cones can provide an effective apical seal similar to 0.02 gutta-percha cones.

## Introduction

The ultimate objective of root canal therapy is to create a complete seal throughout the root canal system; from the coronal opening to the apical termination. To achieve this seal, the root canal system should be prepared chemo-mechanically to attain a sufficient shape and size to facilitate filling of the root canal (1).

Nickel-Titanium (NiTi) rotary instruments which are used for root canal preparation with a crown-down technique, prepare the root canal system with the same standard as the traditional technique (2). These instruments have gained rapid acceptance due to advantages of increased speed in canal preparation and decreased operator fatigue (3). 

Obturation of root canals being prepared with NiTi instruments may be performed by different techniques *e.g.* thermo-plasticized or lateral compaction. Cold lateral compaction of gutta-percha is the common method for root canal obturation (4). A 0.02 mm/mm tapered master gutta-percha cone and numerous accessory cones are generally utilized. Recently, 0.04 and 0.06 mm/mm tapered gutta-percha cones have been manufactured to correlate with the various tapers of rotary NiTi instruments. Filling canals prepared with NiTi instruments with a corresponding tapered gutta-percha master cone and lateral compaction technique may increase clinical efficiency, and has gained widespread acceptance with clinicians (5). Obturation performed with 0.04 and 0.06 gutta-percha cones reduce the number of accessory points required in cold lateral condensation and increases efficiency and speed when compared with 0.02 gutta-percha cones (6).

Nagas *et al.* found that the use of matched-taper gutta-percha points in canals prepared with tapered rotary instruments may improve the bond strength of root canal filling materials to the root canal walls (7).

The use of a master cone with larger taper also increases the volume of gutta-percha, and therefore reducing the amount of sealer required between accessory cones; a desirable feature that improves the three-dimensional obturation of the root canal (8).

Zhao *et al*. study showed significant difference between the leakage of 0.02 taper gutta-percha groups and 0.06 taper groups and concluded that an improved apical seal was obtained when using the same tapered gutta-percha cone with the root canal size (9).

Pérez Heredia *et al*. compared the apical seal in canals obturated using Ultrafil® 3D injectable low-temperature thermoplasticized gutta-percha with cold lateral condensation using either 0.06 or a 0.02 mm/mm tapered master cone. They found that these techniques created equally good apical seal in curved canals (10). 

The purpose of this study was to compare the leakage between 0.02 taper and 0.04 tapered gutta-percha master cones.

## Materials and Methods

A total of forty-four extracted human single rooted teeth with mature apices and straight canals were selected. All samples were cleaned and stored in normal saline solution. The clinical crown of each tooth was cut at the cemento-enamel junction using a high-speed fissure bur and water spray; then, the length of roots was adjusted to approximately 16 mm. 

The working length of each canal was determined by placing a #15 K-file (Maillefer, Dentsply, Switzerland) into the canal until it exited from the apical foramen. The working length was determined to be 1 mm shorter than this length. The roots were instrumented by crown-down technique with 0.06 taper rotary NiTi instruments (Easy RaCe, FKG, Switzerland) meticulously following the manufacturer's instruction. During instrumentation, the canals were irrigated with 2.5% NaOCl. The smear layer was removed with a final flush of 1 mL of 17% EDTA solution, followed by 3 mL of 5.25% NaOCl. Finally, all remnants of EDTA and NaOCl were removed by rinsing the root canals with 3 mL of normal saline; the canals were then dried with paper points.

The teeth were randomly divided into two experimental groups of 20 teeth each and two control groups of 2 teeth each. 

In group 1, the teeth were obturated by the cold lateral compaction technique using 0.02 tapered master cones. After placing AH26 sealer (Dentsply, DeTrey, Konstanz, Germany) in the canal, a size 30, 0.02 gutta-percha cones was coated with sealer and placed into the canal at the working length. Accessory gutta-percha cons (size 20, 0.02 gutta-percha) were then added until the spreader could not be inserted beyond the coronal one third of the canal. A heated instrument was used to cut the gutta-percha cones at canal orifice level and the coronal mass was compacted using a plugger. 

In group 2, the teeth were obturated with 0.04 tapered master cones, AH26 sealer and the same method lateral compaction.

In positive control group the roots were prepared without obturation and in negative control group the teeth were obturated and then all surfaces of the roots were coated with two layers of nail varnish.

Specimens were stored at 37^°^C in 100% humidity for 72 hours to allow sealers to completely set; subsequently, each tooth was placed into a device designed for measuring apical microleakage by fluid transport.

**Figure 1:**
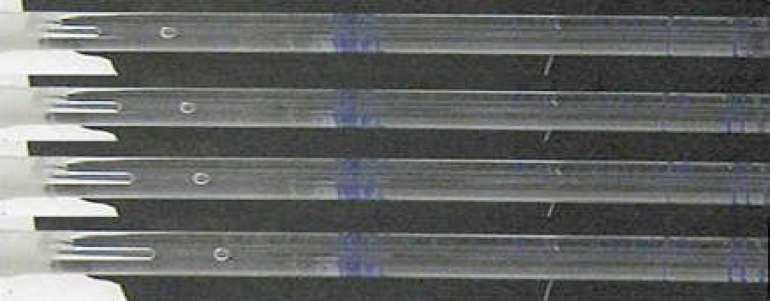
Digital photographs taken at 2-min intervals demonstrated bubble movement

The volume of the fluid transport was measured by observing the movement of the air bubble. The observation was conducted 30 seconds after pressure use for localization of the bubble and then digital photographs were taken for 8 minutes at 2 minutes intervals (Figure1). Finally, the designed software was used to measure the bubble movement. Data were calculated in μL/min/cmH_2_O and analyzed with student t-test. 

## Results

The positive controls showed immediate transportation of the air bubble, whereas the negative controls showed no microleakage. The mean microleakage for the 0.02 and 0.04 experimental groups were 0.195 and 0.23 respectively. 

Table 1 illustrates the mean microleakage values in each experimental group. There were no significant differences between the mean microleakage of two experimental groups (P=0.558).

## Discussion

Previous studies have indicated that RaCe rotary file instruments were able to remove debris while maintaining the original canal curvature (11); thus in the present study shaping and filing was carried out with RaCe instruments to test this hypothesis. 

Several methods have been employed to evaluate the sealing ability of root canal filling materials; these include dye penetration, radioisotopes, fluorometrics, electrochemical, scanning electron microscopic examination, root clarification and fluid filtration techniques (12). The fluid filtration technique is one of the best techniques for the quantitative measurement of microleakage of filling materials and root canal apical seal as fluid filtration models can be re-measured as samples are not destroyed (13). In this study, fluid filtration method was used to measure apical microleakage of canals.

Results of this study showed no statistical significant differences between the mean microleakage in the experimental groups. However, the mean leakage was slightly greater, but not significantly in canals filled with 0.04 tapered master cones. 

Hembrough *et al.* compared the obturation quality and efficiency of lateral condensation with different tapered gutta-percha cones and showed that 0.06 tapered gutta-percha cones required less accessory points than 0.02 taper cones, and therefore they were more efficient for lateral compaction. However, the obturation quality was not significantly different (14).

**Table 1:** The mean, minimum and maximum microleakage of two groups

**Taper**	**Mean**	**SD**	**Min**	**Max**
0.02	0.1954	0.1090	0.0298	0.4307
0.04	0.2300	0.2481	0.0233	1.0065
Total	0.02140	0.1949	0.0233	1.0065

*T=0.59*      *P=0.558*

Pérez Heredia *et al.* also compared the apical seal in canals obturated using thermoplasticized

gutta-percha or cold lateral condensation with either 0.06 or 0.02 tapered master cone and found that these techniques were equally effective in creating a good apical seal (10). 

The slightly greater apical microleakage in canals filled with 0.04 tapered master cones in this study can be attributed to the lower spreader insertion depth in these canals. Gutta-percha cones with greater taper do not allow spreader insertion within 1 mm of the working length (5,15). Allison *et al*. found that teeth in which a spreader tip could be inserted within 1 mm of the working length had considerably less apical leakage than teeth in which the distance between the spreader tip and working length was greater (16). 

Bal *et al.* found no significant differences in apical leakage among the canals that were obturated with 0.06 or 0.02 tapered gutta-percha cones. However, the mean leakage was greater in canals filled with 0.06 tapered master cone than in those filled with 0.02 tapered master cone, which was attributed to the lower penetration depth of the spreader in the 0.06 tapered master cone group (5).

Master cones with a larger taper have closer proximity to the prepared canal walls and thus may avert the spreader insertion to 1-2 mm of the working length. This could result in the inadequate compaction of the master cone in apical portion of the curved canals and deficiency in the seal. Further studies on teeth with more complex anatomy are required.

## Conclusion

Under the condition of this study, cold lateral compaction with 0.04 tapered gutta-percha master cones can create as effective apical seal as 0.02 tapered master cones in simple canals prepared with NiTi rotary instruments.
